# Becoming a neurosurgeon in the United Kingdom: A road map for medical students and early career doctors

**DOI:** 10.1016/j.amsu.2022.103387

**Published:** 2022-02-20

**Authors:** Damilola Alexander Jesuyajolu

**Affiliations:** First Graceland Hospitals, Abijo, Nigeria, Km 43, Lekki-Epe Expressway, Abijo-Ajah, Lagos State, Nigeria

## Abstract

Neurosurgery is one of the most sought-after specialties worldwide. It is one of the most competitive specialties in the United Kingdom. The entire process takes 8 years and the recruitment process aims to select the best of candidates. Not much has been said about the entire selection process from the online application to the interview process, especially with the unprecedented changes brought about by the coronavirus pandemic. There needed to be a roadmap of the entire process, from start to finish, to enable the medical students and early career doctors to make an informed decision, as well as prepare beforehand to meet the criteria that have been set out. A balanced perspective is needed as well, to highlight the drawbacks of pursuing this arduous training specialty in the United Kingdom. This article builds on existing information and throws more light on the application, interview, and the challenges faced by doctors in training. It also shows the challenges the international medical graduate might face during the application process in terms of the shortlisting matrix as well as during training. This article offers advice to all doctors willing to specialize in neurosurgery and highlights what they can do to improve their chances. It sheds more light on the interview process so that shortlisted doctors can know where to focus on during the interviews. Most importantly, by describing the challenges that may be encountered as doctors in training under the NHS, it aims to give a balanced view. This will enable the would-be candidate to make a well-informed choice.

## Highlights

• Neurosurgical training is highly competitive in the UK.
• The process involves an online application (building of portfolio) and writing the multi-specialist recruitment exam.
• International medical graduates have some challenges with some of the portfolio domains.
• Important domains highlighted as well as things that can be done to improve each domain.
• An overview of what takes place in the interview.

## 1 Introduction

Neurosurgery is without a doubt, one of the most coveted specialties worldwide [1]. Advanced surgical practices, good remuneration, and a seemingly stable healthcare system make the United Kingdom (UK) a prime destination for graduates seeking to advance their careers in neurosurgery. The National Health Service (NHS) is home to one of the most competitive neurosurgical programs with a high competitive ratio [2]. Furthermore, the number of available training posts has been decreasing yearly, which is likely due to the increasing number of post-training fellows who find it difficult to secure consultant posts after completing their neurosurgery residency training programs [3]. This dwindling number makes it more competitive for graduates to secure a neurosurgical training role. In 2019, a decision was made to place medical jobs, including surgery, on the shortage of occupation list. This decision made it possible for international medical graduates (IMGs) to apply for roles at the same time as those who studied in the UK [4]. There are challenges, however, that may be faced by medical students and early career doctors upon entering the NHS workforce. This article will help to create a road map of the neurosurgical recruitment process to allow early planning for interested medical students and graduates. More importantly, by describing the challenges that may be encountered as a doctor under the NHS, it aims to give a balanced view. One that would enable the would-be candidate to make a well-informed choice.

## 2 Neurosurgical training in the United Kingdom

The Neurosurgical training program runs for 8 years, from ST1 to ST8. The trainees are expected to have completed the foundation competencies before entering into the program [5]. Evidence of this includes the Foundation Programme Certificate of Completion (FPCC) awarded to those who complete foundation year programs and a Certificate of Readiness to Enter Specialty Training (CREST) form for those who did not. The first three years allow the candidate to focus on core neuroscience training, as well as learn the fundamentals of basic surgery. The next three years require the candidate to build on existing knowledge and develop competencies in the management of a wide variety of neurosurgical conditions. In the final two years, the neurosurgeons would be fully committed to theatre duties and would have more time to consolidate the practical aspects of the training program [6]. Upon completion of the program, if from ST1, they are awarded a CCT. Trainees can also enter into the program from an ST2 level, but it is even more competitive, and the number of slots available per year is few. Only one slot was available in 2021 [7]. After training, the fellows are employed to serve as consultants in the NHS.

### 2.1 Application process

Applications are open every year, and candidates are expected to apply through a central system called ORIEL. Eligible candidates are then long-listed and are invited to take the Multi-Specialty Recruitment Assessment. The applications are given scores through a shortlisting matrix, and this score is added to the results from the MSRA. The total score determines whether a candidate would be invited for an interview [8].

### 2.2 Challenges as an IMG

The challenges faced by an IMG cut across all domains. While the exam to be shortlisted, the MSRA, offers an objective method of getting shortlisted for an interview, it only makes up 40% of the shortlisting score. The larger percentage of the score is made up of points that are accrued across more than 10 domains [8]. Each of these domains presents a particularly unique challenge to the international medical graduate. Highlighting this challenge, however, will help to show areas where prospective IMGs wishing to be neurosurgeons can work to increase their chances.

### 2.3 The neurosurgery shortlisting matrix

This matrix highlights a list of domains, all of which are given scores from 0 to 3 with the total maximum obtainable score as at 2022 being 42. While there is no clear definition as to how each of these domains can be scored, a comparison with the domain scorings of other specialties' shortlisting matrices will help highlight what might be relevant to fulfill the neurosurgery shortlisting criteria [9].

### 2.4 What the matrix entails

#### 2.4.1 Audit and quality improvement projects

Audits and quality improvement projects are valued in the NHS, and medical students who train in the UK are encouraged to actively participate in such projects. Clinical audits are important in healthcare [10]. In other countries, medical students do not have the same privileges. For many, their first encounter with audits is during application for NHS roles after passing the Professional and Linguistic Assessment Board Exams (PLAB). Would-be candidates need to participate actively in clinical audits and quality improvement projects as these would attract maximum points.

#### 2.4.2 Higher degrees and intercalated degrees

Medical schools often operate differently, and in medical schools in the United Kingdom, it is often straightforward to enroll in an intercalated degree, or finish a primary degree and then enroll in a medical school. Maximum points are usually awarded for added degrees with good performance. Students in African countries, Nigeria for instance, do not have the same privilege [11]. Understanding this early on can allow international graduates to seek such opportunities in their medical schools.

#### 2.4.3 Research, publications, and presentations

The research culture is very poor among IMGs in their home countries. Most doctors, despite being aware of research, choose not to participate during their undergraduate years. Most only gain research experience during their final year project [11]. Active participation in research projects which will be peer-reviewed and published would help to gain the necessary points to increase the chances of being shortlisted for interviews [12]. Furthermore, these research projects can be presented at regional and international meetings either orally or with the use of posters at conferences and they can be published in PubMed-indexed journals. Most surgical bodies and relevant associations offer prizes and awards to the best presentations submitted, so it can also serve as a source of awards.

#### 2.4.4 Awards and prizes

Awards and prizes can be gotten at undergraduate and postgraduate levels. Graduating top of the class in medical school is a feat and is a prized source of points during shortlisting. It requires a lot of commitment, discipline, and time management, all of which are desirable features. Essay prizes and abstract prizes gotten from regional and international bodies also contribute to getting shortlisted for this role. At a post-graduate level, getting prizes and awards is laudable as they often require extra effort and planning. At undergraduate and postgraduate levels, getting awards will score maximum points [12].

#### 2.4.5 Clinical courses

Surgical courses present a very good way to show commitment to surgical training. Courses like Basic Surgical Skills, Advanced Trauma Life Support, and Care of the Critically Ill Neurosurgical Patient can go a long way [13]. These courses are usually expensive; nonetheless, they can be paid for when the incoming doctors begin working with the NHS. Some trusts offer a paid study leave which will go a long way in providing time and money to enroll in these courses.

#### 2.4.6 Teaching

Teaching is a very important skill that every doctor must have. The ability to teach the next generation is a skill that is valued by the NHS. Most doctors would have taught at some point in time, but they often fail to collect feedback and letters from their departments as proof of the teaching activities they have carried out. Such evidence can prove useful and can give high scores in this domain. Acquiring a PGdip in teaching alongside organizing a regional teaching program is likely to gain maximum marks. Another easier but less rewarding option is to take a ‘train the trainer’ course. Being aware of these criteria can allow the prospective candidates to quickly collate evidence to keep in their portfolio.

### 2.5 The interview

Candidates who meet the minimum number of points would be invited for an interview [8]. At the interview, there are two major kinds of stations.

#### 2.5.1 Presentation/portfolio station

Candidates are usually given a short presentation to make which usually involves their interest in neurosurgery while the examiners take a look at the portfolio [14].

#### 2.5.2 Clinical station

Different kinds of stations could be presented before the candidate, each designed to test the candidate's ability to work in a neurosurgery department.

##### 2.5.2.1 Clinical neurosurgical scenarios

Candidates are to work their way through clinical scenarios to identify differentials, identify radiologic findings and propose management plans.

##### 2.5.2.2 Simulation stations

These stations emphasize communication skills as they simulate real-life scenarios with actors posing as patients.

##### 2.5.2.3 Telephone stations

Candidates are tested on their ability to take and make referrals in a neurosurgery department.

##### 2.5.2.4 Management stations

The examiner may describe challenging work scenarios to explore skills such as time management and dealing with conflict or stress.

##### 2.5.2.5 Practical skill stations

The practical station consists of 3 separate tasks, each assessed by an examiner. Practical and psychomotor skills are very important for the would-be neurosurgeon and as such, this particular section is designed to show the examiner how proficient the candidate is in that area [14].

Successful candidates are sent offers of employment and are ready for resumption.

## 3 The challenges of neurosurgery training in the UK

### 3.1 Unemployment

The Neurosurgical training process takes 8 long arduous years in the United Kingdom, which is at least a year more than the length of time it takes to become a specialist in the United States (7 years) [15], and significantly more than the time it takes in the rest of Europe (about 4–6 years) [16]. Despite this long duration, many residents who become fellows are left without consultant posts. Between 2015 and 2018 alone, the amount of post-CCT neurosurgery fellows increased remarkably from 26 to 43, and that figure is likely to continue to rise. The UK Neurosurgery Workforce Report 2020 by the Society of British Neurosurgeons highlights the reduction in the number of training posts in order to curb this effect. Unless adequate workforce planning is done, the situation is unlikely to change.

### 3.2 Quality of training

Neurosurgeons in other countries like the United States, despite having less number of years in training programs, are often better exposed when compared to their colleagues who trained in the United Kingdom. Research, for example, is one of the core components of the training given in the United States. In many competent centers in the United States, there is a dedicated period of 1–2 years of research [17]. This is a tad different from the training in the United Kingdom whose training is a bit directed towards service delivery.

### 3.3 Racism and bias

Institutional racism yet remains a debated issue in the NHS. It has been said that the Black and Minority Ethnic group of doctors, despite constituting a figure of about 29% of all UK doctors, make up a staggering 42% of the complaints made by employers. An important case includes that of Mr. Kareem, a consultant urologist, for which it was proven that he was discriminated against because of his race [18]. Doctors from the Black and Minority Ethnic group are more likely to be referred to the General Medical Council for fitness to practice concerns. This occurred frequently such that the GMC led a research project to understand why this is so [19]. Ethnicity and bias remain a factor in the NHS, and independent research carried out for the General Medical Council shows that ethnicity affects the prospects of future doctors in the United Kingdom [20].

### 3.4 Lack of support

Understaffing is a frequent problem that occurs in many NHS institutions, and this often affects junior doctors negatively. The lack of support for these doctors makes the situation worse. When Chris Day, a junior intensive doctor disclosed the poor staffing situation to the management of his trust and Health Education England (HEE), rather than act on the complaint, the event kick-started a series of events that ultimately ended at an employment tribunal [21]. Understaffing was one of the central problems that led to the Mid Staffs Hospital scandal, one that was estimated to have caused the death of between 400 and 1200 patients as a result of the ensuing poor care. A day-by-day diary of a junior doctor in the NHS gives more insight as to the lack of support, rota gaps, and burdensome work that can befall a junior doctor in the system [22]. The NHS workforce crisis has worsened over time, with the pandemic causing staffing shortages on an unprecedented scale [23], and on top of that, staff, especially doctors still plan to quit after the pandemic is over [24].

## 4 Conclusion

Adequate preparation and planning are needed by the medical student and early-career doctor when deciding on the path to a neurosurgery residency in the UK. This article would help to give a road map as well as a balanced perspective for students and doctors, both UK and foreign-trained, aspiring to be neurosurgeons.

## Provenance and peer review

Not commissioned, externally peer reviewed.

## Funding

No funding was required.

## Ethical approval

Article did not require ethical approval.

## Consent

Article did not involve patients or volunteers.

## Author contribution

The entire article was undertaken by the sole author. Damilola Alexander Jesuyajolu.

## Guarantor

DAMILOLA ALEXANDER JESUYAJOLU, SOLE AUTHOR.

## Declaration of competing interest

There are no conflicts of interest.
